# Structural and Quantitative Analysis of Three C-Glycosylflavones by Variable Temperature Proton Quantitative Nuclear Magnetic Resonance

**DOI:** 10.1155/2017/4934309

**Published:** 2017-01-23

**Authors:** Jing Liu, Yang Liu, Zhong Dai, Lan He, Shuangcheng Ma

**Affiliations:** National Institutes for Food and Drug Control, Beijing 100050, China

## Abstract

Quantitative nuclear magnetic resonance is a powerful tool in drug analysis because of its speed, precision, and efficiency. In present study, the application of variable temperature proton quantitative nuclear magnetic resonance (VT-^1^H-qNMR) for the calibration of three C-glycosylflavones including orientin, isoorientin, and schaftoside as reference substances was reported. Since there was conformational equilibrium due to the restricted rotation around the C(sp^3^)-C(sp^2^) bond in C-glycosylflavones, the conformational behaviors were investigated by VT-NMR and verified by molecular mechanics (MM) calculation. The VT-^1^H-qNMR method was validated including the linearity, limit of quantification, precision, and stability. The results were consistent with those obtained from mass balance approach. VT-^1^H-qNMR can be deployed as an effective tool in analyzing C-glycosylflavones.

## 1. Introduction

Quantitative nuclear magnetic resonance (qNMR) spectrometry was first reported in the 1960s. This method has been widely applied to various fields such as drug analysis, reference substances quality control, and natural products due to its high speed and precision [1–4]. qNMR technique has been adopted in all major national pharmacopoeias including US pharmacopeia, European pharmacopeia, Japanese pharmacopeia, and Chinese pharmacopeia [5–9]. For qNMR, the resonance signal is directly proportional to the number of resonant nuclei. Therefore, this approach has various advantages such as no need for reference substances or large amount of organic solvents.

**Figure 1:**
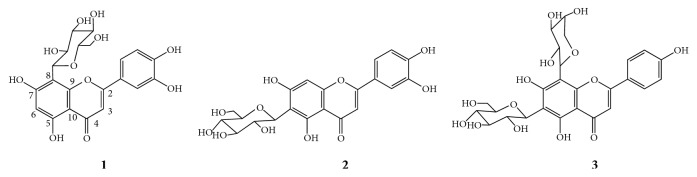
Structures of orientin (**1**), isoorientin (**2**), and schaftoside (**3**).

C-glycosylflavone is a unique type of natural product with various pharmacological effects including scavenging free radicals and protecting myocardial ischemia [10, 11]. Although qNMR technique has been widely used in characterization of reference substances of different structure types, there is no report on the C-glycosylflavones due to poor response signal from proton NMR. Herein, orientin (**1**), isoorientin (**2**), and schaftoside (**3**), three common flavone C-glycosides with sugar moieties at C_6_ and/or C_8_ (Figure 1), were selected for ^1^H qNMR study. For this type of compounds, the restricted rotation around the C(sp^3^)-C(sp^2^) bond results in the coexistence of rotational isomers which might complicate the NMR spectrum. Since increasing temperature will eliminate the carbon-carbon bond rotation barrier, the conformational equilibrium of three C-glycosylflavones was directly characterized by variable temperature NMR (VT-NMR). Meanwhile, the conformational behaviors of the three C-glycosylflavones were investigated by using molecular mechanics 2 (MM2) calculation. Variable temperature proton quantitative nuclear magnetic resonance (VT-^1^H-qNMR) was also applied to directly determine the content of orientin, isoorientin, and schaftoside for the first time. The results are consistent with the data from mass balance method. VT-^1^H-qNMR method is an effective approach to achieve satisfactory result for C-glycosylflavones.

## 2. Materials and Methods

### 2.1. Materials and Analyte Preparations

Orientin (97.9%), isoorientin (94.0%), and schaftoside (93.1%) (determined by mass balance method) were from National Institutes for Food and Drug Control, Beijing, China; 1,4-dinitrobenzene was purchased from TCI chemicals (99.0%, Lot. 3EUXH-JB). DMSO-*d*_6_ was from Sigma (99.9%, St. Louis, USA).

Test samples and internal standard 1,4-dinitrobenzene were dissolved in DMSO-*d*_6_ to produce a concentration of about 0.04 mol/mL and 0.03 mol/mL, respectively. For linearity, different concentration of schaftoside ranging from 5.01 to 30.09 mg was dissolved in 1.0 mL DMSO-*d*_6_.

### 2.2. Instrument Conditions

The ^1^H NMR spectra were acquired at 298 K or 348 K using a Bruker Ascend 500 spectrometer with a BBO probe at 500.15 MHz. For qNMR, the following parameters were applied: 30° pulse angle, spectral width equal to 20 ppm, acquisition time equal to 3.28 s, receiver gain equal to 162, O1P equal to 6.15 ppm, 64 K data points, 16 scans, and relaxation time *D*1 equal to 20 s. 90° pulse calibration was conducted daily to make sure of the performance of NMR spectrometer.

### 2.3. Processing Parameters

Data was processed on MestReNova 6.1.1 with 0.3 Hz exponential apodization applied to FID. Manual phase correction and signal integrations were performed corresponding to the IS signals and sample signals. ^1^H NMR shift was referenced to the solvent signal of DMSO-d_6_.

### 2.4. Content Calculation Formula

The content of sample was calculated by the following formula:

(1) $$\begin{array}{c} W_{s}\left(\;\%\;\right)=\frac{\left(A_{s}/n_{s}\right)\times M_{s}\times m_{r}}{\left(A_{r}/n_{r}\right)\times M_{r}\times m_{s}}\times P_{r}\times 100\%, \end{array}$$

where *A*_*s*_ and *A*_*r*_ are the signal response of the samples and IS, *n*_*s*_ and *n*_*r*_ are the numbers of spin atoms in the analyte and IS, *M*_*s*_ is the molecular weight of samples (448.38 g/mol for orientin and isoorientin, 564.49 g/mol for schaftoside), *M*_*r*_ is the molecular weight of IS (168.11 g/mol), *m*_*s*_ and *m*_*r*_ are the masses of the analytes and IS, and *P*_*r*_ is the purity of the IS.

## 3. Result and Discussion

### 3.1. Experiments Parameters

For pulse flip angle, most of the qualitative proton NMR and some of qNMR experiments are performed with 30° pulse. Our group use 30° in our routine ^1^H-qNMR experiments and get reasonable results. Although 90° pulse will give better *S*/*N* than 30°, 30° in VT-^1^H-qNMR can partly represent the real circumstance in using ^1^H-qNMR.

As a critical parameter in VT-^1^H-qNMR experiments, relaxation time (*D*1) should be more than 5 times that of longitudinal relaxation (*T*1) to allow the activated proton to return to equilibrium status. The *T*1 values were determined by an inversion recovery method. *T*1 of the internal standard and analyte signal was found to be 1.5 s and 3.9 s, respectively. So *D*1 was set as 20 s in this study.

### 3.2. Conformational Analysis

Atropisomers occur when rotation around a C-C single bond is hindered by the rotational energy barrier. For most C(sp^2^)-C(sp^3^) single bond, the rotational energy barrier is low, and the isomerism could not be observed at room temperature. For some C-glycosylflavones, NMR spectra acquired under room temperature showed signals corresponding to atropisomers at different ratio due to the high rotational energy barrier. The phenomenon was verified by means of variable temperature NMR experiments and theoretical MM2 calculations [12–16].

**Figure 2:**
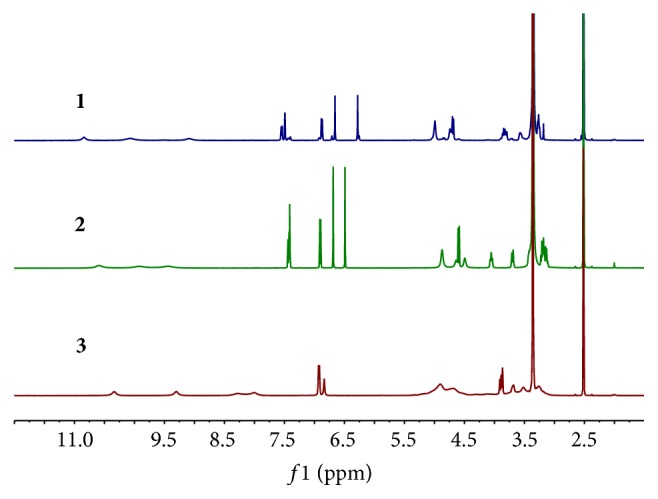
^1^H NMR spectra of orientin (**1**), isoorientin (**2**), and schaftoside (**3**) (298 K).

**Figure 3:**
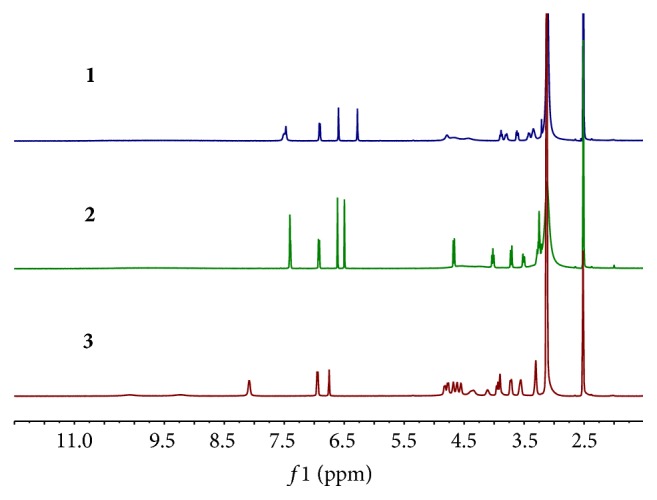
^1^H NMR spectra of orientin (**1**), isoorientin (**2**), and schaftoside (**3**) (348 K).

During our study, the ^1^H NMR spectra acquired at 298 K (Figure 2) showed some impurity signals around the aromatic and anomeric protons for orientin and isoorientin, respectively. And the spectrum for schaftoside presented some signals not splitting well. Considering the structure similarity with those reported [12–16], the above phenomenon was inferred from restricted rotation. Therefore, VT-^1^H-qNMR experiments were carried out in order to verify the deduction. Increasing the temperature from 298 K to 348 K altered the ^1^H NMR spectra. The aromatic and anomeric proton signals appear to undergo coalescence at 348 K for orientin and isoorientin as shown in Figure 3. Also the spectrum presented signals splitting well, especially those around the aromatic region for schaftoside (Figure 3).

**Figure 4:**
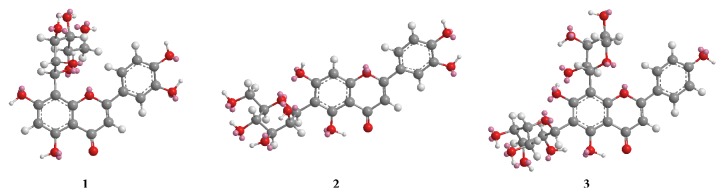
MM2 computed structures of the lowest energy conformers of orientin (**1**), isoorientin (**2**), and schaftoside (**3**).

It was demonstrated that steric hindrance was the main effect that influences the rotational isomerism [16]. In this study, the difference between the isomers of orientin and isoorientin is the different position of the glucosyl substituent. Due to the bigger substitute at C_9_ compared to that at C_5_, it would be subjected to greater rotation hindrance for orientin compared to isoorientin. As a result, it is obvious that the ^1^H NMR spectrum of orientin presented impurity signals around the aromatic region corresponding to the rotational isomer. For the C-diglycosylflavone of schaftoside, it was obviously more difficult to overcome the rotation barrier. The conformational analysis for three compounds was performed* via* molecular mechanics using the MM force field in ChemBio 3D Ultra software (Figure 4). Since structures of the three compounds were different, the absolute energy was useless for comparison, and the energy difference between conformers of the same compound is meaningful. The calculated energy difference for orientin, isoorientin, and schaftoside was 9.222, 5.429, and 34.809 kcal/mol, respectively. Bigger energy difference represents the higher rotational barrier.

### 3.3. Selection of Sample Signals and IS Signals

**Figure 5:**
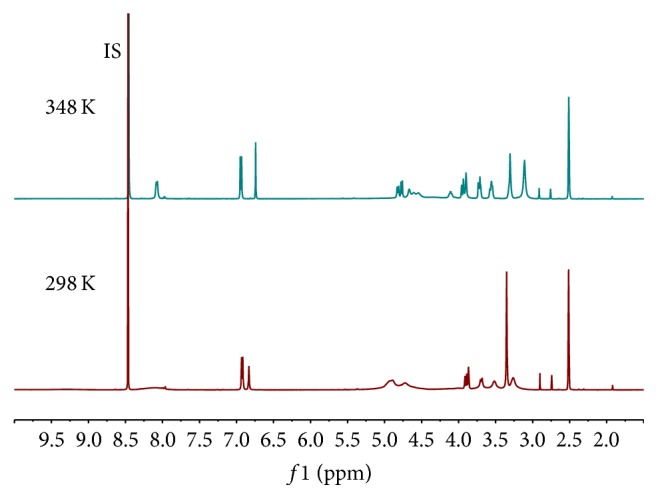
VT-^1^H-qNMR spectra of schaftoside and internal standard (IS).

1,4-Dinitrobenzene was selected as the internal standard during the experiment due to the following reasons: high solubility and the chemical shift of the aromatic protons provide a well-separated signal (*δ* 8.4) without any interference with orientin, isoorientin, and schaftoside in the integration region. In our experiments, the singlet signal at *δ* 6.6 for orientin and isoorientin and *δ* 6.7 for schaftoside were used for quantification, respectively (Figure 5).

### 3.4. Method Validation

#### 3.4.1. Linearity and Range

Schaftoside was used as a model compound to validate VT-^1^H-qNMR.

The solutions for linearity test were prepared by dissolving different amount of schaftoside and IS to the required concentrations. The calibration curve was drawn for the ratio of sample to IS (*X*) versus the ratio of selected sample signal to IS signal (*Y*) (Table 1). The correction coefficient showed it had good linearity within 5.01~30.09 mg/mL concentration ranges (*Y* = 0.068*X* + 0.007, *R*^2^ = 0.998).

#### 3.4.2. Limit of Quantification (LOQ)

It is reported that the signal to noise ratio (*S*/*N*) should be more than 150 in quantitative experiments to produce good quantification results [16]. Here, *S*/*N* = 150 was used to calculate LOQ. LOQ for schaftoside is 2.45 mg/mL.

#### 3.4.3. Precision, Repeatability, and Stability

Precision tests were carried out by characterizing the same sample six times. And repeatability was achieved by characterizing five independent solutions containing both the sample and IS. Both RSD indicated the good accuracy of the method. The stability was assessed by analyzing one sample at 1-, 2-, 4-, 6-, and 8-hour interval. The results indicated that schaftoside was stable after 8 hours in solution.

Method validation results were summarized (Table 1).

### 3.5. Comparison Results from VT-^1^H-qNMR with Mass Balance Method (Table 2)

The established VT-^1^H-qNMR method was applied for the calibration of orientin, isoorientin, and schaftoside. Also the mass balance approach was used for calculation [17]. Table 2 shows that the results of the three C-glycoflavones by VT-^1^H-qNMR are similar to data from mass balance method.

## 4. Conclusions

Contents of some flavone C-glycosides cannot be achieved due to the existence of isomers. This study developed a reliable VT-^1^H-qNMR method to determine the content of three common flavone C-glycosides: orientin, isoorientin, and schaftoside. Comparing the qNMR method with the mass balance approach, the contents of orientin, isoorientin, and schaftoside were similar. VT-^1^H-qNMR method could be complementary to the mass balance approach for the value assignment of the reference substances. This technology is a powerful tool in drug quality control.

## Figures and Tables

**Table 1:** Linearity, range, and precision of schaftoside calculated by VT-^1^H-qNMR (348 K).

Linearity and range				Precision				Repeatability	
Sample	*m*_*r*_ (mg/mL)	*m*_*s*_ (mg/mL)	*A*_*s*_/*A*_*r*_	Sample	*m*_*r*_ (mg/mL)	*m*_*s*_ (mg/mL)	*W*_*s*_ (%)	No.	*W*_*s*_ (%)
1	5.48	5.01	0.0759	1	4.64	19.46	93.51	1	94.42
2	5.44	10.40	0.1334	2	5.17	20.00	94.17	2	93.91
3	4.78	14.82	0.2201	3	5.34	20.11	93.89	3	94.15
4	5.84	18.98	0.2296	4	4.74	19.97	93.07	4	93.58
5	5.28	30.09	0.4015	5	4.73	19.98	94.16	5	93.99
				6	5.84	18.98	94.42		
*R* ^2^	0.998				—				—
RSD (%)	—				0.53				0.33

**Table 2:** Content from VT-^1^H-qNMR (348 K) and mass balance method (%).

	Orientin	Isoorientin	Schaftoside
VT-^1^H-qNMR	98.1 (RSD 0.49%)	93.5 (RSD 0.49%)	93.9 (RSD 0.53%)
Mass balance	97.9	94.0	93.1
